# Aerobic capacity and disease activity in children, adolescents and young adults with juvenile idiopathic arthritis (JIA)

**DOI:** 10.1186/1546-0096-10-25

**Published:** 2012-08-20

**Authors:** Philomine A van Pelt, Tim Takken, Marco van Brussel, Mirjam de Witte, Aike A Kruize, Nico M Wulffraat

**Affiliations:** 1 Department of Paediatric Immunology, Wilhelmina Children’s Hospital, University Medical Center Utrecht, Utrecht, Netherlands; 2 Department of Rheumatology, Erasmus Medical Center, University Medical Center Rotterdam, Rotterdam, The Netherlands; 3 Child Development & Exercise Center, Wilhelmina Children’s Hospital, UMC Utrecht, Utrecht, The Netherlands; 4 Department of Rheumatology & Clinical Immunology, University Medical Center Utrecht, Utrecht, The Netherlands

**Keywords:** Juvenile idiopathic arthritis, Aerobic capacity, Disease activity, Remission, Transition, Adolescents

## Abstract

**Background:**

As patients with juvenile idiopathic arthritis (JIA) progress into adulthood, long-term outcome is determined by disease activity, physical and psychosocial development. Decreased aerobic capacity may play a critical role in health-related outcomes in JIA, since it has been linked with cardiovascular morbidity and mortality in late adulthood. The objectives of the current study are to examine the aerobic capacity and its relation to parameters of disease activity in children, adolescents and young adults with JIA.

**Methods:**

Sixty-three patients with JIA (aged 10–27 years) were cross sectional studied regarding their aerobic capacity and correlations were made to demographic, disease-related variables, and medication utilization. in a cross-sectional study group of 63 patients of all subtypes. Patients were divided in three age groups, 10–13 years; 14–17 years and 18–27 years.

**Results:**

Reduced aerobic capacity is found in clinical remission as well as active disease in all subtypes and all age groups. Aerobic capacity is more impaired in active disease shown by DAS 28, JADAS 27, ESR and serum thrombocyte counts. Lower haemoglobin has a negative impact. Long-term used medication including methotrexate and corticosteroids didn’t influence outcome. There is no association with current sports participation.

**Conclusion:**

Reduced aerobic capacity is present in adolescents and young adults with JIA, both in active disease and in patients with remission. Measures of aerobic capacity may serve as important outcome measure in JIA.

## Background

Although Juvenile Idiopathic Arthritis (JIA) is generally thought to have a favourable outcome [1], in reality its course may be less propitious. At adult age, persistent disease activity and functional impairment have been reported in more than half of the patients [2-6].Moreover, disease related chronic inflammation may cause protein loss and fat accumulation [7], increasing the risk for obesity and cardiovascular disease in this population [8]. Systemic inflammation, immune dysfunction and its treatment [9-12], might also contribute to the increased mortality rate in JIA [10,11].

In addition to these disease-related factors [5], factors influencing morbidity in the general population must also be considered as they are likely to have heightened effect on patients with JIA. For example, low aerobic capacity, a marker of physical fitness, may play a critical role in health-related outcomes in JIA since it has been linked with an increased risk of developing chronic diseases including cardiovascular diseases, diabetes, cancer, hypertension, obesity, depression and osteoporosis [13], as well as an increased risk of mortality in adults [14].

Physical fitness is a multidimensional concept defined as a set of attributes that people possess or achieve that relate to the ability to perform physical activity [15]. It is comprised of skill-related (e.g. agility, coordination), health-related (e.g. cardiovascular capacity), and physiological components [16]. In adults, the level of physical fitness is a powerful predictor of mortality in both healthy and disease states [17,18]. The gold standard for assessing physical fitness is the aerobic capacity, commonly defined as the maximal or peak volume of oxygen uptake (VO_2peak_) during an incremental exercise test to exhaustion. VO_2peak_ is expressed either as an absolute value in liters of oxygen per minute (l/min) or as a relative value in milliliters of oxygen per kilogram of bodyweight per minute (ml/kg/min). The relative value is frequently used to compare the fitness levels of patients with a chronic disease including a wide range of ages, body sizes and disease severity.

Aerobic capacity has been shown to be impaired in both younger children and adolescents with JIA [19-21]. These impairments may be associated with a combination of the condition-related pathophysiology itself, its treatment (e.g. medications), hypoactivity and subsequent deconditioning [22]. Other potential contributing factors to reduced aerobic capacity in JIA may include anemia, reduced cardiac output, and muscular dysfunction [20].

Patients with JIA may suffer from normocytic hypochromic anaemia, which implies lower oxygen transport capacity of the blood, and hence, impaired oxygen transport to the muscles. A recent study of children with JIA revealed no significant differences in cardiac output during exercise between the JIA group and healthy controls [23]. Muscle atrophy, however, is a common finding in patients with JIA that may be linked to inactivity, medication utilization (including glucocorticoids) and disease activity [24].

Although its usage is generally related to disease activity and severity, medication itself can indirectly affect aerobic capacity in patients with JIA through its impact on body composition. For example, pharmacological doses of glucocorticoids are associated with protein loss, bone loss and insulin resistance in both hepatic and peripheral tissues, leading to impaired carbohydrate metabolism [18,25].An inverse correlation was found between duration of MTX treatment and respiratory muscle function in children with systemic and polyarticular JIA, possibly explained by a more severe disease course being associated with long-term MTX treatment [26] In contrast to observed findings in adult rheumatoid arthritis (RA) patients [27], in children with JIA, impairment of lung function by MTX is rare [28,29]. Effects of anti-TNF therapy on aerobic capacity are not known yet.

To our knowledge, data on aerobic capacity in relation to the disease activity in patients with JIA are scarce [30]. The aims of our current study are therefore to examine the aerobic capacity and its relation to parameters of disease activity in children, adolescents and young adults with JIA.

## Methods

All consecutive patients between 2005 and 2008 with JIA (as classified by the International League Against Rheumatism criteria, ILAR{Petty, Southwood, et al. 2004 163/id [31]) between the ages of 10 to 27 years were asked to participate in an observational longitudinal cohort study, investigating clinical parameters and aerobic capacity in children, adolescents and young adults with JIA over a 3-year period. A total of 63 patients provided informed consent. The results of baseline assessments are described in the present study. All patients were seen in the out-patient clinics of the paediatric or adult departments of rheumatology & immunology at the University Medical Center (UMC) Utrecht. Patients were divided into three age groups, 10–13 years, 14–17 years and 18–27 years, with the latter patient group recruited from the adult rheumatology department. The middle age group (14–17 years) will be transferred to adult health care over the course of the three-year follow-up study. The Medical-Ethics Committee of the UMC Utrecht approved all study procedures.

Demographic variables, disease characteristics and medication utilization (past and current) were recorded for all patients (Table 1). Functional ability was assessed with the validated Dutch version of the Childhood Health Assessment Questionnaire [32-34]. Patients were asked about which sports and leisure activities were performed and the time consuming per week in a general interview [35]. Physical examination included a general assessment. Standard laboratory values including Erythrocyte Sedimentation Rate (ESR), C - reactive protein (CRP), thrombocytes and haemoglobin were obtained from a blood sample collected at the outpatient clinic. Haemoglobin counts were used to classify anaemia based on international reference values for in children and adolescents [36]. 

**Table 1:** Demographic and disease related variables

**N = number of patients**	**Age 10–13 yrs N = 33**	**Age 14–17 yrs N = 18**	**Age 18–27 yrs N = 12**	**All patients N = 63**
Boys (N)	13	6	3	22
Girls (N)	20	12	9	41
Subtype JIA N (%)				
Systemic	3 (9.1)	4 (22.2)	1 (8.3)	8 (12.7)
Oligo persistent	3 (9.1)	0 (0)	0(0)	3 (4.8)
Oligo extended	9 (27.3)	3 (16.7)	3 (25.0)	15 (23.8)
Polyarticular RF+	3 (9.1)	2 (11.1)	2 (16.7)	7 (11.1)
Polyarticular RF-	14 (42.4)	8 (44.4)	5 (41.7)	27 (42.9)
Enthesitis related	0 (0)	1 (5.6)	1 (8.3)	2 (3.2)
Psoriatic arthritis	1 (3.0)	0 (0)	0 (0)	1 (1.6)
Disease Duration in years (IQR)	5.5 (6.0)	10.5 (6.0)	10.0 (18.0)	8.0 (7.0)
CHAQ (IQR)	1,6 (0.8)	1,4 (0.3)	1,4 (0.6)_	1,5 (0.8)
Sports performance (hours/week, IOR)	2.0 (3.0)	3.0 (8.0)	2.5 (6.0)	2.0 (3.0)
BMI kg/m^2^ (IQR)	17.9 (3.3)	21.3 (4.5)	19.8 (3.9)	18.8 (4.7)
Z-score VO_2peak_ (IQR)	−0.9 (1.5)	−0.8 (1.6)	−0.6 (1.7)	−0.8 (1.3)**
Z-score VO_2peak_/kg (IQR)	−1.6 (1.7)	−1.5 (2.1)	−1.0 (2.1)	−1.5 (1.9)**
ESR mm (IQR)	8.0 (10.0)	9.5 (16.0)	7.0 (14.0)	8.0 (13.0)
CRP mg/l (IQR)	2.0 (5.0)	6.0 (23.0)	6.0 (5.0)	5.5 (6.0)
Haemoglobin mmol/l (IQR)	8.0 (1.0)	7.6 (1.0)	8.7 (1.3)	7.9 (1.3)
Thrombocytes 10.9/l (IQR)	315 (102)	273 (102)	290 (93)	298 (83)
DAS28 (IQR)	1.7 (1.1)	1.8 (1.9)	1.5 (1.6)	1.6 (1.2)
JADAS 27 (IQR)	3.6 (7.1)	3.0 (22.9)	2.6 (17.1)	2.9 (9.0)
VAS patient (IQR)	0.2 (2.3)	0.5 (3.1)	0.5 (.6)	0.5 (2.3)
Number of swollen joints (IQR)	0.0 (2.0)	0.0 (3.0)	0.0 (3.0)	0.0 (4.4)
Number of tender joints (IQR)	0.0 (3.5)	0.0 (4.0)	0.0 (3.8)	0.0 (4.1)
Number of limited joints (IQR)	1.5 (4.0)	2.0 (5.0)	2.5 (5.0)	2.0 (4.0)

All variables are expressed as median values unless otherwise stated.

Abbreviations

JIA: Juvenile Idiopathic Arthritis; IQR: interquartile range; CHAQ: Childhood Health Assessment Questionnaire; Sports performance ref: [35] BMI: Body Mass Index; Z-score_:_ standard deviation of the individual patient compared with values from age- and sex- related historical Dutch controls [37]; VO_2peak abs_: highest oxygen uptake in liters of oxygen per minute (l/min) during maximum exercise test in l/min; VO_2peak_/kg : highest oxygen uptake in milliliters of oxygen per kilogram of bodyweight per minute (ml/min/kg) during maximum exercise; ESR: Erythrocyte Sedimentation Rate; CRP: C-Reactive Protein; DAS28: Disease Activity Score of 28 joints; JADAS 27: Juvenile Arthritis Disease Activity Score of 27 joints; VAS patient: Visual Analogue Scale of General Health.

** p < 0.01 significant difference compared to healthy age and sex related individuals.

### Aerobic capacity

Aerobic capacity (VO_2peak_) was assessed using a graded cardiopulmonary exercise test (CPET) to volitional exhaustion performed on an electronically braked cycle ergometer (Lode examiner, Lode BV, Groningen, the Netherlands). The seat height was adjusted to the patient’s comfort. Patients began cycling at a workload of 0 Watts and this increased by 20 Watts every minute until they were no longer able to continue cycling due to volitional exhaustion, despite strong verbal encouragement. Patients breathed through a mouthpiece, which was connected to a calibrated metabolic cart (Oxycon Pro, Care Fusion, Houten, the Netherlands). Expired gases were collected and analysed for breath-by-breath minute ventilation (Ve), oxygen uptake (VO_2_), carbon dioxide exhalation (VCO_2_) and respiratory exchange ratio (RER; =VCO_2_/VO_2_) using conventional equations. The highest achieved oxygen uptake averaged over a 30-second period was taken as VO_2peak._ Heart rate (HR) was measured continuously via 3-lead ECG. To allow comparison of aerobic capacity with healthy controls, standardized z-scores were determined using: Z-scores *= [(observed VO*_*2peak*_*- predicted VO*_*2peak*_*)/SD]* where predicted VO_2peak_ values were obtained from a database of established values for age- and gender- related Dutch controls [37]. A z-score of zero represents a VO_2_ similar to that of healthy controls, while a score of ±1.5 is a clinical important difference from healthy controls.

### Disease activity

A number of indicators were used to assess disease activity including the total number of swollen joints, number of painful joints, the number of joints with limited range of motion, number of active joints (defined as joints with swelling and/or pain during motion), the Juvenile Arthritis Disease Activity Score (JADAS 27) [38]and the Disease Activity Index 28 (DAS28) [39]. All of the examinations were performed by an experienced (paediatric) rheumatologist. Furthermore, a global assessment was performed by the patient (well being; VAS patient 0-100 mm) as well by a (paediatric) rheumatologist (disease activity; PGA 0-100 mm). Given that patients were recruited from both the paediatric and adult clinics, disease activity scores commonly used in both the paediatric and adult departments were computed. In adult Rheumatoid Arthritis (RA) patients, the DAS28 is a commonly used and validated tool to measure disease activity [39]. This is a compound score of the number of a total of swollen joints (out of 28), the number of a total of painful joints (out of 28), assessment of patient’s general health and the erythrocyte sedimentation rate (ESR). Three compound disease activity scores were recently introduced in paediatric rheumatology: Juvenile Arthritis Disease Activity Score (JADAS) 10, JADAS 27 and JADAS 71 [38]. We utilised the JADAS 27 compound score because of its feasibility. Moreover, statistical performance of JADAS 27 is comparable to JADAS 71, while the construct validity of JADAS 10 is poor compared to JADAS 71 and JADAS 27 [38]. The JADAS 27 may present an advantage over the DAS 28 because it includes measures for the cervical spine, hips and ankles, joints which are often affected in (adult) patients with JIA.

### Medication use

Past and current medication utilization was obtained from a review of the patient’s medical chart.

### Statistical analysis

All statistical analyses were performed using Statistical Package for Social Science (SPSS) version 15.0 for windows (SPSS Inc., Chicago, IL) version 15.0 for Windows. Because patient data were non normally distributed non-parametric tests were used to analyse the demographic variables and compare the results of the cardiopulmonary exercise tests (Mann–Whitney and Kruskal-Wallis where appropriate). Spearman’s Correlation test was used for determining the correlations between VO_2peak_ and disease activity markers and standard laboratory values. The level of statistical significance was set at P < 0.05.

## Results

### Demographics

Demographic and disease related characteristics are presented in Table 1. In total, 22 boys and 41 girls participated in the study. The longest disease durations were seen in the oldest age group. No significant differences were noted between age groups with respect to disease duration, body mass index (BMI), disease activity markers as measured by number of swollen and tender joints, DAS28, JADAS 27 and laboratory values.

### Aerobic capacity

**Figure 1:**
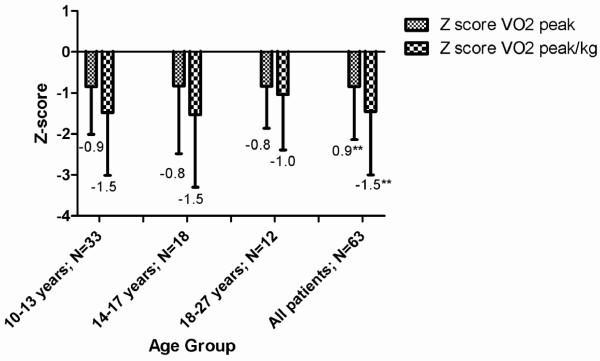
** Aerobic capacity (expressed as median Z-score of VO**_**2peak**_** and VO**_**2peak**_**/kg) in relation to age in adolescents and young adults with JIA.** Z scores are compared to age and sex related healthy controls (p-values <0.01). Abbreviations: Z-score_:_ standard deviation of the individual patient compared with values from age- and sex- related historical Dutch controls [37]; VO_2peak abs_: highest oxygen uptake in liters of oxygen per minute (l/min) during maximum exercise test in l/min; VO_2peak_/kg_l_ : highest oxygen uptake in milliliters of oxygen per kilogram of bodyweight per minute (ml/min/kg) during maximum exercise.

The median peak heart rate was 187 beats per minute (Interquartile range [IQR] 17), indicating an adequate level of maximum achieved exercise. The median aerobic capacity in all patients with JIA was considerably lower compared to healthy peers, with a median z-score of −0.8 (p < 0.01) for absolute VO_2peak_ (L/min) and −1.5 (p < 0.01) for relative VO_2peak_/kg (ml/kg/min) in all patients. VO_2peak_ did not differ significantly by gender, disease duration or JIA sub-type; therefore all patients were merged for further analysis. There were no statistical significant differences in the z-scores for absolute and relative VO_2peak_ between the different age groups (Figure 1).

### Aerobic capacity and disease activity

Spearman’s correlation coefficient revealed a significant negative correlation between the number of swollen joints and absolute VO_2peak_ z-score, but not with relative VO_2peak_ z-score (Table 2). No significant correlations were seen between VO_2peak_ values and the number of painful joints (Table 2). Both disease severity compound scores (DAS28 and JADAS 27) showed significant moderately strong negative associations with all VO_2peak_ values (Table 2).

**Table 2:** Median values (IQR) of Disease activity markers and Spearman’s Correlation Coefficient values for Aerobic Capacity (expressed by absolute and relative VO2 peak) (level of significance:* indicates significance < 0.05; ** indicates significance <0.01)

	**Median (IQR)**	**Correlation Z-score VO**_**2peak **_**(p -value)**	**Correlation Z–score VO**_**2peak**_**/kg (p -value)**
Number of swollen joints in lower extremities	0.0 (0)	−0.3 (**)	−0.1
number of swollen joints in upper and lower extremities	0.0 (2)	−0.3 (**)	−0.1
Number of painful joints lower extremities	0.0 (2)	−0.1	−0.1
number of painful joints in upper and lower extremities	0.0 (4.0)	−0.2	−0.1
number of limited joints in upper and lower extremities	2.0 (4.0))	−0.3 (*)	−0.3 (*)
Active joint count	0.0 (0)	−0.3 (**)	−0.2
VAS patient	0.5 (2.6)	−0.3 (*)	−0.3 (*)
DAS28	1.6 (1.2)	−0.4 (**)	−0.4 (**)
JADAS 27	2.9 (9.0)	−0.3(**)	−0.4(**)

Abbreviations

IQR: interquartile range, Z-score_:_ standard deviation of the individual patient compared with values from age- and sex- related historical Dutch controls(37); VO_2peak abs_: highest oxygen uptake in liters of oxygen per minute (l/min) during maximum exercise test in l/min; VO_2peak_/kg_l_ : highest oxygen uptake in milliliters of oxygen per kilogram of bodyweight per minute (ml/min/kg) during maximum exercise; VAS patient: Visual Analogue Scale of General Health (0-100 mm); DAS28: Disease Activity Score of 28 joints; JADAS 27: Juvenile Arthritis Disease Activity Score of 27 joints.

**Figure 2:**
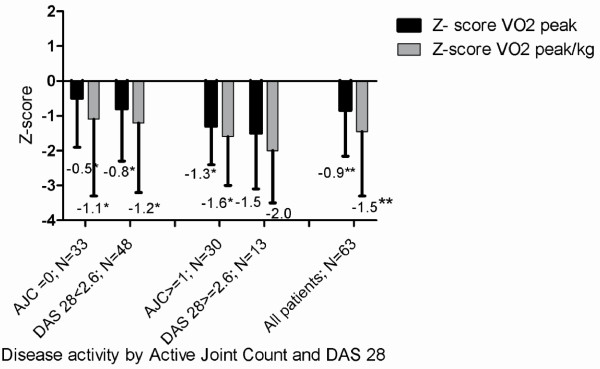
** Aerobic capacity (expressed as Z-score of VO**_**2peak**_** and VO**_**2peak**_** /kg) in relation to disease activity in adolescents and young adults with JIA in remission and with active disease.** Z scores are compared to age and sex matched healthy controls (* p-values <0.05; **p-values <0.01). Abbreviations: Z-score_:_ standard deviation of the individual patient compared with values from age- and sex- related historical Dutch controls [37]; VO_2peak abs_: highest oxygen uptake in liters of oxygen per minute (l/min) during maximum exercise test in l/min; VO_2peak_/kg_l_ : highest oxygen uptake in milliliters of oxygen per kilogram of bodyweight per minute (ml/min/kg) during maximum exercise; AJC: Active Joint Count; DAS28: Disease Activity Score of 28 joints.

A substantial proportion of the patients presented with low disease activity as reflected by low median numbers of swollen and painful joints (Table 2). The patients were divided into 2 groups based on disease activity status, these included patients in remission or inactive disease (defined by Wallace et al. [40], active joint count zero; DAS28 ≤2.6) and a patient group with disease activity (active joint count one and higher; DAS28 > 2.6) [40,41]. Between these patient groups, significant differences as measured by active joint count and DAS28 score were seen (Figure 2). 

Significant negative correlations were observed between laboratory parameters reflecting disease activity (ESR, thrombocytes) and the aerobic capacity (Table 3). Conversely, no relationship was seen between C-reactive protein, an acute phase protein and VO_2peak_ (Table 3). We found a positive significant correlation between serum haemoglobin and the relative VO_2peak_/kg in boys as well in the total patient group. In girls, a positive significant correlation between serum haemoglobin and the absolute VO_2peak_ (p < 0.05, Table 3) was observed. Lower VO_2peak_ and VO_2peak_/kg values were noted in patients with anaemia compared to those who were not anaemic (p < 0.05).

**Table 3:** Median (IQR) Standard Laboratory Values and Spearman’s Correlation Coefficients for Aerobic Capacity (expressed by absolute and relative VO2 peak) (level of significance:* indicates significance < 0.05; ** indicates significance <0.01)

	**Median (IQR)**	**Correlation with Z-score VO**_**2peak **_**(p -value)**	**Correlation Z-score VO**_**2peak**_**/kg (p -value)**
ESR (mm)	8.0 (13)	−0.3 (*)	−0.5 (**)
CRP (mg/l)	5.5 (6)	−0.2	−0.2
Thrombocytes (10.9/l)	302 (110)	−0.4 (**)	−0.5(**)
Haemoglobin (mmol/l) boys	8.1 (1.2)	+0.0	+0.5 (*)
Haemoglobin (mmol/l) girls	7.8 (1.1)	+0.3 (*)	+0.2

Abbreviations

IQR: Interquartile Range; Z-score_:_ standard deviation of the individual patient compared with values from age- and sex- related historical Dutch controls(37); VO_2peak abs_: highest oxygen uptake in liters of oxygen per minute (l/min) during maximum exercise test in l/min; VO_2peak_/kg_l_ : highest oxygen uptake in milliliters of oxygen per kilogram of bodyweight per minute (ml/min/kg) during maximum exercise; ESR: Erythrocyte Sedimentation Rate (ref 2-12 mm in 1 hour); CRP: C-Reactive Protein (ref 0–10 milligram per litre); thrombocytes (ref 150–450 10.9 per litre); Haemoglobin boys (ref: age group 10-12 yrs: 6.6-.8.4 millimol per liter age 12–18 yrs 6.9-9.1 millimol per liter; age >18 yrs: 8.6-10.5 millimol per liter); Hemoglobin girls (ref: age 10–12 yrs 6.6-8.4 millimol per liter; age 12–18 yrs 6.8-8.3 millimol per liter; age >18 yrs:7.5-9.5 millimol per liter).

### Aerobic capacity and use of disease modifying anti-rheumatic drugs (DMARD)

Thirteen of our patients were not using any DMARD at the time of the testing, 38 patients were using one DMARD, 11 patients were using a combination of two DMARD’s and only 1 patient was on a combination therapy of three DMARD’s. Non-parametric tests revealed no significant differences in aerobic capacity between these patients. No significant correlations were found between current use of DMARD and absolute and relative VO_2peak_ values. When looking at historical DMARD use, only use of anti-Tumor Necrosing Factor biologicals in 10 patients was significantly associated with lower absolute and relative VO_2peak_ values (p < 0.05). Duration of current and formerly MTX use did not affect aerobic capacity.

A total of 6 patients were currently using corticosteroids at the time of testing, while 10 patients had used them the past and 47 patients never used corticosteroids. Neither current nor former corticosteroid use was significantly related to aerobic capacity.

## Discussion

Although a favorable long-term outcome is seen in about half of the patients with JIA [1], results of the current study highlight a significant impairment in aerobic capacity, with a median z score of −1.5 in all patients (children, adolescents and young adults with JIA. Although disease duration and DMARD use were expected to have a negative influence on aerobic capacity, no significant correlations, with the exception of past biological use were seen between these variables.

Despite the overall low median disease activity in our patient group, representing the general favorable disease outcome, aerobic capacity was still significantly reduced in patients with JIA compared to healthy controls. Significant impairments in aerobic capacity have previously been reported in youth with other chronic diseases including Crohn’ disease and dermatomyositis [42-44]. A significant correlation of disease activity with aerobic capacity was found (Table 2), the strongest correlations were seen for the compound scores DAS28 and JADAS27. When considering separate items, aerobic capacity was significantly related to the number of swollen, limited, and active joints, but not to the number of painful joints. Apparently, joint-pain is a negligible limiting factor in reaching a maximum of exercise levels.

When the study population was divided by disease activity status, (remission or inactive disease [40] versus active disease) significant differences in aerobic capacity were observed (Figure 2). Since a lower aerobic capacity was also noted in patients in remission compared with healthy controls, one might hypothesize that current indicators of disease activity in JIA are not always sufficient to predict impaired aerobic capacity. As low aerobic capacity is linked with an increased risk of developing other chronic diseases including cardiovascular diseases, diabetes, cancer, hypertension, obesity, depression and osteoporosis [13], as well as an increased risk of mortality in adults [14], this current finding is of importance for the quality of life of all patients with JIA.

Significantly negative correlations were observed between ESR, thrombocytes and both absolute and relative aerobic capacity, suggesting that disease activity and systemic inflammation may negatively affect aerobic capacity. Extensive studies on the role of inflammatory cytokines (e.g. TNF-α) in maintaining the balance between muscle protein synthesis and degradation [7] have lead to the concept of *rheumatoid cachexia*, a condition in which muscle protein degradation is favored over synthesis resulting in muscle wasting, impaired muscle strength and a concomitant increase of fat mass. Body mass index was used as an indirect parameter of body composition in the current study, but did not reveal any significant relationship with aerobic capacity. Although it has been described a common complication in JIA [45], we did not find a significant growth retardation (median length 50^th^ percentile; median weight 49^th^ percentile) in our population.

Future studies in patients with JIA might also consider the inclusion of detailed determination of body composition (muscle mass, fat mass and bone mass) next to muscle strength testing.

This is the first study in patients with JIA to show lower aerobic capacity in individuals with lower serum hemoglobin .This correlation may be explained by a lower oxygen transport capacity to the muscles. In chronic conditions like end-stage kidney disease, anemia is thought to at least partly explain the observed reduction in aerobic capacity [46]. Anemia is often considered a reflection of disease activity [47]*,* suggesting that perhaps the observed correlation with aerobic capacity is due to disease activity, rather than anaemia per se; however, this relationship remained significant when the data were corrected for disease activity.

In our study, no evidence for the influence of any DMARD or glucocorticoids intake on aerobic capacity was found, and also duration of MTX treatment was not correlated with VO_2peak._ Although in earlier days MTX treatment was limited to more severe disease course, in our study population, MTX is used in all subtypes of JIA and in almost all patients (61 out of 63 patients). Only past anti-TNF biological therapy appeared to negatively influence aerobic capacity; this may be related to the fact that anti-TNF in the Netherlands is restricted to patients with a poly-articular disease course after failure of treatment with, or untreatable side effects of high dose MTX. This suggests that a more severe disease course, rather than biological therapy, per se, is associated with a lower aerobic capacity. It is important to note that despite our finding of similar disease activity among all JIA subtypes and DMARD utilization, generally patients with a poly-articular and systemic JIA are considered to have a more severe disease course.

In longstanding JIA muscle atrophy, decreased muscle strength and lower extremity deformities might serve to limit aerobic capacity. Moreover much like adults with RA, patients with JIA may have low levels of physical activity (hypo activity) [22], highlighting the need for more rigorous training programs in this population to prevent the increased risk of developing chronic diseases like cardiovascular disease and diabetes [13,48,49]. In the present study, approximately 73% of the patients reported participating in sport more than 12 times a year, compared with 68% in general population [35]. In our study, sport participation was not significantly correlated with aerobic capacity (results not shown).

We realize that the current patient group represents a select group of patients that may not be reflective of the general pediatric and young adult rheumatology population. More specifically, since the patients in our study, are at least 10 years of age, the percentage of persistent oligo-articular patients is lower than the proportion typically seen in the pediatric outpatient clinic. Despite this patient selection, it is remarkable that a negative correlation was observed between disease activity and aerobic capacity, which persisted into adulthood. Furthermore it is also important to note that although a number of correlations presented were only moderately strong, they remained significant and were consistent across several markers that reflect disease activity, namely DAS 28, JADAS 27, and laboratory markers like ESR, thrombocytes and hemoglobin.

## Conclusions

In conclusion, our results demonstrate that children, adolescents and young adults with JIA suffer from reduced aerobic capacity. This impairment in aerobic capacity was seen in patients with active disease as well as those in remission. Further, we report that aerobic capacity is negatively correlated to disease activity, as measured by physical examination as well as several laboratory parameters. Given that poor aerobic capacity has been linked with a number of healthy complications, these findings highlight the need for longstanding follow up into adulthood of patients with JIA, even if disease activity is low or in remission.

## Competing interests

The authors declare that they have no competing interests.

## Authors’ contributions

PvP participated in design of the study, acquisition of data, statistical analysis, and was responsible for analysis of the results and drafting of the manuscript. TT participated in design of the study, the performance of clinical examinations, and in analysis of the results and drafting and revision of the manuscript. MvB participated in the performance of clinical examinations and revise of the manuscript. MdW helped in the acquisition of data and statistical analysis. AAK contributed to design of the study and revision of the manuscript. NMW participated in design of the study and revision of the manuscript. AAK and NMW shared last authorship. All authors read and approved the final manuscript.
